# Current Strategies to Enhance Delivery of Drugs across the Blood–Brain Barrier

**DOI:** 10.3390/pharmaceutics14050987

**Published:** 2022-05-04

**Authors:** Raluca Ioana Teleanu, Manuela Daniela Preda, Adelina-Gabriela Niculescu, Oana Vladâcenco, Crina Ioana Radu, Alexandru Mihai Grumezescu, Daniel Mihai Teleanu

**Affiliations:** 1 Department of Pediatric Neurology, “Dr. Victor Gomoiu” Children’s Hospital, 022102 Bucharest, Romania; raluca.teleanu@umfcd.ro (R.I.T.); oana-aurelia.vladacenco@drd.umfcd.ro (O.V.); 2 “Carol Davila” University of Medicine and Pharmacy, 020021 Bucharest, Romania; daniel.teleanu@umfcd.ro; 3 Department of Science and Engineering of Oxide Materials and Nanomaterials, Faculty of Applied Chemistry and Materials Science, Politehnica University of Bucharest, 011061 Bucharest, Romania; manuela.preda@stud.fim.upb.ro (M.D.P.); adelina.niculescu@upb.ro (A.-G.N.); 4 Department of Neurosurgery (I), Bucharest University Emergency Hospital, 050098 Bucharest, Romania; crina.radu95@gmail.com; 5 Research Institute of the University of Bucharest—ICUB, University of Bucharest, 050657 Bucharest, Romania; 6 Academy of Romanian Scientists, Ilfov No. 3, 050044 Bucharest, Romania

**Keywords:** blood–brain barrier, medication delivery techniques, nanoparticles

## Abstract

The blood–brain barrier (BBB) has shown to be a significant obstacle to brain medication delivery. The BBB in a healthy brain is a diffusion barrier that prevents most substances from passing from the blood to the brain; only tiny molecules can pass across the BBB. The BBB is disturbed in specific pathological illnesses such as stroke, diabetes, seizures, multiple sclerosis, Parkinson’s disease, and Alzheimer’s disease. The goal of this study is to offer a general overview of current brain medication delivery techniques and associated topics from the last five years. It is anticipated that this review will stimulate readers to look into new ways to deliver medications to the brain. Following an introduction of the construction and function of the BBB in both healthy and pathological conditions, this review revisits certain contested questions, such as whether nanoparticles may cross the BBB on their own and if medications are selectively delivered to the brain by deliberately targeted nanoparticles. Current non-nanoparticle options are also discussed, including drug delivery via the permeable BBB under pathological circumstances and the use of non-invasive approaches to improve brain medication absorption.

## 1. Introduction

Brain diseases, such as central nervous system (CNS) disorders and brain cancers, are the most common, debilitating, and underserved diseases [1]. As the population of seniors and patients with CNS disorders grows over the next 20 years, global drug development for brain diseases must accelerate. However, drug development for brain diseases has the lowest success rates compared to other therapeutic areas. CNS drugs typically take much longer to develop than non-CNS drugs. Because of the complexity of the brain, side effects, and the blood–brain barrier, clinical trials of CNS drugs are difficult [2].

Aside from the complexity of brain diseases, the lack of efficient technologies for delivering drugs across the BBB stymies CNS drug development. Small molecules and macromolecules are being studied as potential therapeutic agents for various brain diseases [3].

However, only small molecules with a molecular weight up to 400 Da and that are lipid-soluble can cross the BBB; most macromolecules cannot penetrate the brain endothelium [4]. The BBB’s physiological barrier prevents 95% of molecules from progressing toward drug development [5]. Recent research has shown that the BBB is a dynamic interface that regulates access of substances from the blood into the brain [4,5]. These advances highlight the importance of rethinking some concepts about brain drug delivery while also revealing significant opportunities for new drug delivery strategies.

This review discusses recent advances in our understanding of the BBB and its disruption in disease conditions. It focuses on new strategies that have been investigated in the last five years to deliver therapeutic and diagnostic agents to the brain. In addition, the effect of aging on the BBB is analyzed.

## 2. General Background on the Blood–Brain Barrier

The brain barrier is significant to medication delivery to the central nervous system. They are made up of numerous parallel barriers; the two best characterized being the vascular BBB, which is made up mostly of the capillary bed, and the blood-cerebrospinal fluid (blood-CSF) barrier, which is made up primarily of the choroid plexus [6,7].

All tissues and organs in the body rely on blood arteries to provide oxygen and nutrients [7,8]. The blood–brain barrier (BBB) is a set of features that allow the blood vessels that vascularize the CNS to strictly regulate the transit of ions, chemicals, and cells between the blood and the brain [9]. Changes in these barrier properties are a significant component of the pathophysiology and development of numerous neurological illnesses since they allow for optimal neuronal activity while also shielding neural tissue from poisons and pathogens [10]. 

Endothelial cells (ECs), which constitute the walls of blood arteries, veins, and capillaries of the brain, skin, lung, heart, and muscle, have a number of physical, transport, and metabolic features that help to coordinate the physiological barrier. Interactions with diverse vascular, immunological, and brain cells influence these features [11]. Understanding how these various cell types interact to modify barrier properties is crucial to understanding how the brain functions in health and disease.

The blood–brain barrier is a part of the neurovascular unit (NVU) that acts as a blood–brain interface, allowing the CNS to communicate with the rest of the body. The BBB divides the circulatory system from the brain, providing for serum factor and neurotoxic protection as well as transport management. In addition to acting as a physical barrier (due to the presence of specialized tight junctions and other changes that prevent unregulated leakage), the BBB serves as a transport interface (with specific transporters present on luminal and abluminal membranes), a secretory body, and a metabolic barrier (containing and releasing certain enzymes locally) [12].

**Figure 1:**
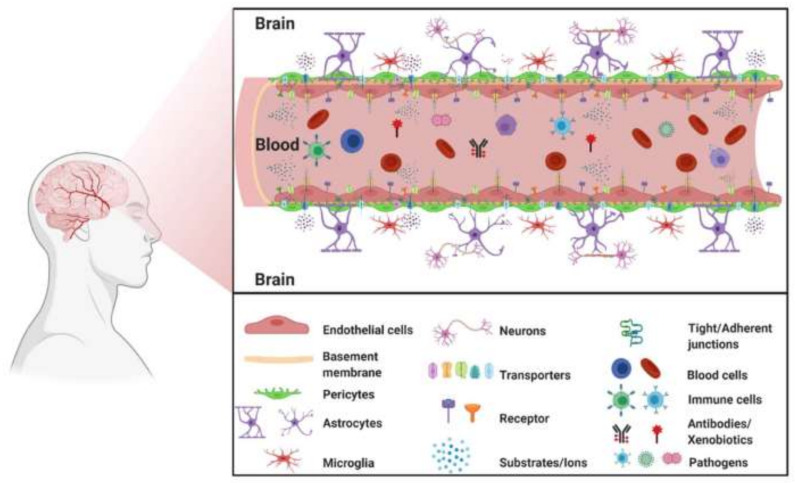
The blood–brain barrier elements. Reprinted from Ref. [14], MDPI, 2021.

The loss of these barrier qualities during neurological illnesses such as stroke, multiple sclerosis (MS), brain traumas, and neurodegenerative disorders is a fundamental component of their pathophysiology and development [13]. BBB disruption can result in ion dysregulation, disrupted signaling homeostasis, and immune cell and molecule entrance into the CNS, all of which can contribute to neuronal dysfunction and degeneration [12]. Figure 1 presents the blood–brain barrier elements.

### 2.1. The Blood–Brain Barrier in a Healthy Brain

The BBB is a diffusion barrier necessary for regular brain function that prevents chemicals from entering the blood and reaching the brain, thus maintaining brain homeostasis. Endothelial cells, pericytes, astrocytes, tight junctions (TJs), neurons, and the basal membrane of the brain form physically tight brain capillaries in the BBB [15]. The absence of fenestrations in brain capillary ECs inhibits the transport of tiny molecules and proteins. Interendothelial junctions connect the ECs to a continuous barrier, preventing water-soluble compounds from penetrating. Pericytes, astrocytes, and the basal membrane surround the ECs, forming the BBB.

Furthermore, efflux transporters are found in brain capillary ECs, which are additional barriers to drugs entering the brain. Interendothelial junctions and protein complexes such as adherens junctions, TJs, and gap junctions primarily govern BBB permeability [16]. Adherens junctions are principally responsible for regulating the permeability of the endothelial barrier. TJs are necessary for the epithelium and ECs to maintain their permeability barriers, which govern tissue homeostasis [17]. Gap junctions, which are made up of six connexin molecules, allow ECs to communicate directly in both electric and chemical terms. The components of the BBB are continually adjusting in response to different physiological changes in the brain, rather than having a set structure [16].

Molecules can pass the BBB through either a paracellular (between neighboring cells) or a transcellular route (through the cells). Ions and solutes use concentration gradients to move across the BBB via passive diffusion in the paracellular route. Passive diffusion, receptor-mediated transport, and transcytosis are part of the transcellular route. In general, passive diffusion is a non-saturable process determined by the molecule’s physicochemical features. Molecular weight, charge, lipid solubility, surface activity, and relative molecule size are all physicochemical parameters that impact BBB permeability [18].

Small lipophilic molecules such as carbon dioxide diffuse passively over the BBB via a transcellular pathway. BBB permeability can be influenced by physiological factors such as efflux transporters (e.g., P-glycoprotein (P-gp)), enzymatic activity, plasma protein binding, and cerebral blood flow [19]. Glucose transporter-1 (GLUT-1), insulin transporter, and transferrin transporter are examples of distinct and highly value receptor-mediated transport routes that allow proteins and peptides to reach the brain [20]. The luminal and abluminal EC membranes exhibit these endogenous transporters. Among these transport systems, receptor-mediated transcytosis has received the most attention in drug delivery into the brain [21]. Greater knowledge of the mechanics of passage over the BBB will aid in the development of innovative medication delivery techniques into the brain.

### 2.2. Blood–Brain Barrier Disruption in Certain Pathological Conditions

**Figure 2:**
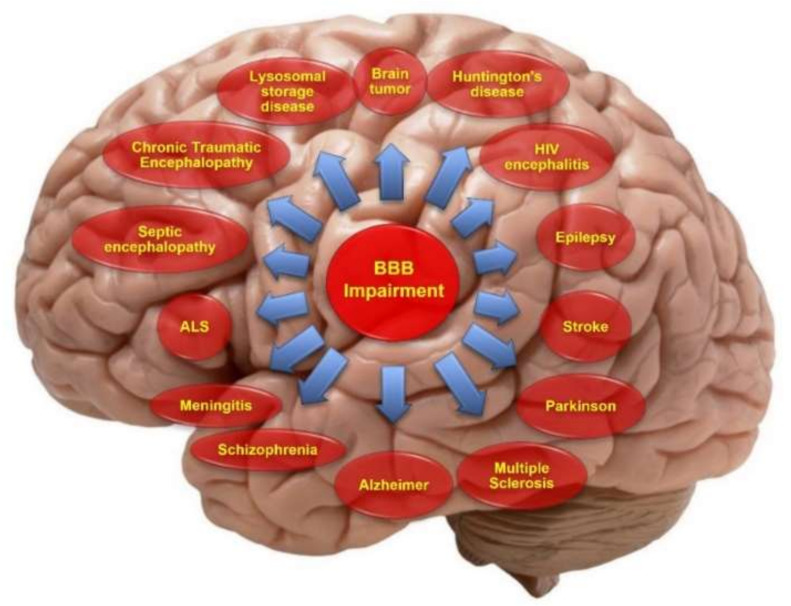
Schematic illustration summarizing some of the major neurological disorders associated with impairment of the BBB. Reprinted from Ref. [25], MDPI, 2021.

Stroke, diabetes, seizures, hypertensive encephalopathy, acquired immunodeficiency syndrome, traumatic brain traumas, multiple sclerosis, Parkinson’s disease (PD), and Alzheimer’s disease (AD) (Figure 2) all disrupt the BBB [22,23,24]. Remodeling the protein complex at interendothelial junctions is a significant cause of BBB collapse in some clinical circumstances.

Shiraishi et al. evaluated magnetic resonance imaging (MRI) contrast agents, gadolinium micelles (Gd-micelles), and gadolinium-diethylenetriamine pentaacetic acid (Gd-DTPA) in rats after intravenous administration. They found a greater contrast signal from Gd-micelles in the ischemic hemisphere than from Gd-DTPA, demonstrating a hyper-permeable BBB under ischemia circumstances [26]. Remodeling the protein complex at interendothelial junctions is a significant cause of BBB collapse in some clinical circumstances. During an ischemic stroke, for example, the BBB becomes more permeable to macromolecules. Because albumin, a large protein molecule, seldom crosses the healthy BBB, it evaluates BBB leakage. In an R6/2 mouse model of Huntington’s disease, albumin-fluorescein isothiocyanate conjugate (FITC-albumin) was found in the brain at early and late disease stages, confirming BBB breakdown under these conditions [27].

The loss of junctional molecules in cholesterol-rich cell membrane areas contributes to increased BBB permeability in multiple sclerosis. Furthermore, altering adherens junctions can drastically alter BBB permeability [28]. As a result, it has been established that connections are disturbed, and as a result, the BBB becomes permeable in some disorders. However, because of several constraints, the degree and time duration of BBB breakdown in each illness is unknown. Multiple sclerosis, for example, is characterized by a transitory or persistent decrease of BBB permeability. Although BBB breakdown may be seen in vivo by injecting a Gd contrast agent, MRI scanning may underestimate the total level of BBB disruption. BBB disruption, on the other hand, is linked to illness consequences. In the case of Alzheimer’s disease, vascular dementia and the disease are usually co-occurring. Along with many contradictory results, it is generally acknowledged that the increase in BBB permeability in certain Alzheimer’s patients is caused by vascular dementia, but not by pure Alzheimer’s disease [29]. Considering the intricacy of mechanisms in CNS illnesses, investigations on BBB disruption in diverse diseases are still in their early stages.

### 2.3. Blood–Brain Tumor Barrier

**Figure 3:**
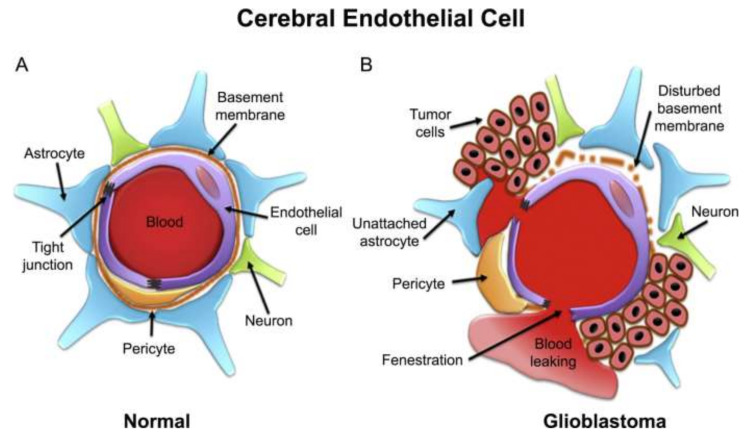
Schematic representation of cerebral capillary showing endothelial cell in (**A**) normal and (**B**) glioblastoma. Reprinted with permission from Ref. [32], Copyright 2020, Elsevier.

The most frequent type of primary brain tumor is glioma [30]. Brain tumor cells mirror the BBB in their early stages due to their rapid proliferation and movement. The BBB is disrupted when tumor cells reach a specific threshold. As a result, the blood–brain tumor barrier (BBTB) is separate from the BBB and is produced by new blood vessels (brain tumor capillary) (Figure 3). In glioblastomas, BBTB permeability is high in the bulk of the tumor but low or non-existent in the periphery [31]. 

As a result, the combination of the BBB and the BBTB creates a significant barrier to brain tumor medication delivery. To circumvent the BBB, strategies such as opening TJs using a hyperosmotic mannitol solution or a chemical (e.g., bradykinin), blocking efflux drug transporters, and receptor-mediated drug delivery systems may be used to improve medication delivery to brain tumors selectively. In addition to passing the BBTB, glioma cells must be targeted. Coating cell-permeable peptides might accomplish this on the surface of nanoparticles. A comprehensive review of peptides for glioma cell targeting may be obtained [33]. This review is mostly concerned with crossing the BBTB.

### 2.4. Approaches to Inhibit ABC Transporters

ATP-binding cassette (ABC) transporters are highly expressed by brain endothelial cells forming the BBB, being involved in maintaining brain homeostasis. Their enhanced activity represents a major obstacle in treating brain diseases, as ABC transporters impede drugs to reach their site of action within CNS [34]. Thus, a discussion on BBB would not be complete without properly addressing the topic of ABC transporters, especially considering the approaches to inhibit these efflux pumps. 

Numerous transporter proteins in the ABC superfamily are involved in expelling compounds utilizing the catalytic energy of ATP. This superfamily actively effluxes substances in humans, as it does in other eukaryotic systems, extruding poisons and medications out of cells [35]. They work with a wide range of substrates, including tiny molecules like ions and molecules, as well as larger, highly organized structures like peptides, lipids, and polysaccharides [36]. In humans, 48 ABC transporters are categorized into seven subfamilies based on their genetic and amino acid structure, from A to G. Furthermore, ABC transporters are thought to hinder pharmacological substances from penetrating into the brain, posing a barrier to therapeutic delivery in neurological illnesses [37].

ABC transporters, which are significant players in the multidrug-resistant phenotype of glioma and other malignancies, efflux several chemotherapeutics [38]. As a result, many efforts have been made to improve tumor drug delivery by using competitive or non-competitive inhibitors, and many P-gp and BCRP inhibitors, such as valspodar [39], dexverapamil [40], tariquidar [41], biricodar [40], and elacridar, have been clinically evaluated for use as adjuvants on chemotherapy to treat non-brain tumors, as well as indirect inhibition by anti-P-gp monoclonal antibodies. Their effectiveness in overcoming the BBB has been tested in both animals and humans. In a rat xenograft model of glioma, for example, greater brain accumulation of erlotinib was reported when it was coadministered with elacridar [42].

The penetration of ^11^C-verapamil was increased when it was given with cyclosporine A in a clinical research on healthy volunteers [43]. However, due to adverse effects such as cardiovascular toxicity of first-generation inhibitors and pharmacokinetic interactions with chemotherapeutics (e.g., inhibition of CYPs450) leading to increased systemic cytotoxicity, ABC transporter inhibition has not been translated into clinical application. Furthermore, several modulators block more than one ABC transporter, such as elacridar and tariquidar, which inhibit both P-gp and BCRP, causing severe side effects such as hazardous chemical buildup in the brain, kidneys, liver, and other organs [44].

LY335979, formerly Zosuquidar trihydrochlorid, was designed as a strong and specific inhibitor of P-gp and was the most advanced compound being tested in the clinics [45]. Nagaya et al. employed zosuquidar in their most recent work to illustrate the influence of P-Glycoprotein–mediated active efflux on drug distribution.

Because the expression levels of P-gp in the BBB of cynomolgus monkeys are similar to those in humans, they were utilized. The effect of a P-gp inhibitor (zosuquidar) on drug CNS penetration in monkeys was also investigated in vivo to determine the influence of P-gp on drug CNS penetration. This work designed a zosuquidar dosing strategy to induce strong P-gp suppression throughout a 24-h period. The findings help them better understand how drugs penetrate the central nervous system in the preclinical stage [46].

But though inhibiting ABC transporters has still not been shown to be effective in clinical drug delivery, they have been a valuable resource in furthering our understanding of ABC transporter function and multidrug resistance in cancer, glioma, and other CNS pathologies, as well as their implications at the blood–brain interface [38].

## 3. Advances in Drug Delivery across the Blood–Brain Barrier

These strategic treatment techniques for circumventing the BBB can be roughly grouped into one or more of the following groups: invasive and non-invasive procedures, as well as other ways. The advantages and limitations of these two procedures are presented in Table 1.

### 3.1. Invasive Approaches

#### 3.1.1. Blood–Brain Barrier Transient Disruption

The use of noxious agents, hyperosmotic solutions, or ultrasounds (mannitol [56], dimethyl sulphoxide [57], ethanol [58], metals [59], glycerol [60], polysorbate-80 [61], X-irradiation [62]) to diminish the brain’s endothelial cells by breaking down tight connections permits diverse substances to circulate into the cerebral tissue. Admittedly, this technique has significant drawbacks: it is not patient-friendly, and it may compromise the integrity and neurobiological functioning of the BBB, potentially resulting in the accumulation of unwanted blood components and neurotoxic, xenobiotic, and exogenous substances, which can cause CNS injury [63].

Electroporation is a recently proposed approach for producing transitory, localized BBB rupture (EP) [64]. During EP, pulsed electrical fields (PEFs) are administered to cells or tissue, causing the electrical potential across the cell membrane to destabilize. Changes in membrane potential cause the formation of nanoscale aqueous holes in the lipid bilayer, increasing the permeability of the cell membrane. If the membrane re-seals, this is referred to as reversible EP; if the PEFs cause cell death, this is referred to as irreversible EP [65]. Earlier studies established the possibility of obtaining EP-induced transitory BBB breakdown in rats after EP treatments using contrast-enhanced T1-weighted MRI [66].

These findings were confirmed in canines, and the electric field threshold for EP-induced BBB breakdown was determined to be in the 500–700 V/cm range in these investigations [67,68]. Bonakdar et al. and Lopez-Quintero et al. revealed that 750,000 low voltage (1–10 V) pulses (90 s pulses at 0.4 ms pulse intervals for 5 min) increased the permeability of bovine aortic endothelial cells in vitro [68]. They discovered higher hydraulic conductivity, which they ascribed to the ZO-1 protein’s disturbing continuity.

#### 3.1.2. Intracerebroventricular and Intrathecal Infusion

These methods include injecting or infusing therapeutic proteins directly into the cerebrospinal fluid via intraventricular infusion (CSF) [69].

The advantages of these approaches over systemic enzyme replacement therapy (ERT) are that they allow for the transport of a greater number of enzymes to the brain and, as a result, do not require the use of enormous quantities of therapeutic medications. Furthermore, these solutions address the issues associated with the short half-life of medicines in circulation while avoiding those associated with systemic exposure and toxicity. A lumbar puncture or an implanted intrathecal medication delivery device can be used to provide intrathecal drugs (IDDD) [70].

ERT administered via intrathecal injection can allocate the recombinant enzyme all across the CNS, allowing it to penetrate brain tissue and start promoting the clearance of aggregated material within the lysosomes, according to statistics from animal models of MPS I, II, and IIIA, as well as other LSDs such as infantile neuronal ceroid lipofuscinosis and Niemann-Pick A [71,72]. In recent years, researchers have concentrated their attention on creating and testing therapeutics for activation of the brain in MPS IIIA, due to the availability of mice [73] and dog models [74] capable of recapitulating the MPS IIIA neuropathological characteristics.

Clinical trials in humans have begun to test the safety and tolerability of recombinant human heparan-N-sulfatase (rhHNS) administered via IDDD in patients with MPS IIIA, pursuing the remarkable results of animal studies that showed that repetitive direct infusion of an absent enzyme via cerebrospinal fluid injection is a successful therapy for pathological changes in the brains of mice and dogs (NCT01155778 and NCT01299727) [22,54]. Similarly, the toxicity of idursulphase, which was formulated for intrathecal injection (idursulphase-IT) on MPS II patients, was investigated via IDDD [75]. Although the findings of these studies motivate more research, clinical implementation of these procedures is regarded as difficult due to the enzymes’ short half-life. Repeated doses with an increased risk of adverse consequences are required to improve effectiveness and raise the possibility of clinical success.

### 3.2. Non-Invasive Approaches

Non-invasive approaches mostly consist of pharmacological tactics capable of altering pharmaceuticals to allow them to pass across the BBB. The primary non-invasive procedures are detailed further below.

#### 3.2.1. Chemical Modification of Drugs

The transvascular approach preceding systemic injection is the primary limitation but the only one to consider for chronic CNS disorders, whether tumoral or neurological [76]. Because many severe neurological disorders do not react to small-molecule treatments, large molecules, therapeutic peptides, inhibitors, or other medications are necessary [77]. As a result, medication delivery to the brain requires methods such as nanoparticles capable of ferrying pharmaceuticals over the BBB. The BBB excludes hydrophilic medications and the vast majority of molecules, although some (but not all) tiny (300 Da) lipophilic molecules may get through via diffusion [78,79]. A medicine can be lipidated to be carried to the brain, however, this strategy has had limited success since increasing lipophilicity may be efficient [80], but it usually comes at the expense of a greater biodistribution. As most transporters are selective, this technique requires that the medication imitate the endogenous ligand.

Following the Maillard process, glycosylation or glycation may improve medication and peptide transport to the brain while enhancing biological stability [81]. Due to better transport, which was likely controlled by adsorptive endocytosis instead of the glucose transporter, and retained bioactivity, Enkephalin-Ser6 b–DGlucose demonstrated higher blood stability and absorption by the brain [82]. EGF (a ligand for differentially expressed EGF receptors in brain cancer) or BDNF (a neurotrophic component) have been linked to a monoclonal antibody against transferrin receptor, resulting in bifunctional molecules that can attach to the BBB transferrin receptor, ferry peptides throughout the vascular wall, and distribute these peptides to the brain [62,63]. The activation of enzyme activities is also important in the BBB, and these functions can be changed by sickness. Aminopeptidase A activity is increased in brain tumors, but aminopeptidase N activity is reduced [83]. These differences are crucial for transporting undamaged molecules to the brain, and enzymatically intact (pro)drugs may be necessary, such as via cyclization, halogenation, methylation, pegylation, or the inclusion of unnatural bonds [84].

#### 3.2.2. Virus-Mediated Distribution across the Blood–Brain Barrier

A viral vector can be delivered via the BBB using one of two methods. The first is transcytosis across brain vascular endothelial cells mediated by receptors [85]; the other is the BBB’s temporary disintegration [86], enabling the vector to reach CNS interstitial regions via paracellular transport.

Traditionally, this has been accomplished by osmotically decreasing the BBB cells via intravenous (IV) infusion of highly concentrated mannitol (25%). This sugar ethanol does not pass through the cell and is excreted through the kidneys fairly quickly. For decades, this technique has been used to facilitate the flow of chemotherapeutic drugs through the BBB to target brain cancers and to reduce intracranial pressure after traumatic brain injury or encephalitis [68,69].

Despite the fact that alternative ways for increasing BBB permeability exist, including adenosine receptor activation and human serum albumin nanoparticles, they still have not yet been employed to distribute viral vectors [70,71].

BBB osmotic rupture regulation controlled by mannitol has been utilized to transfer adenovirus (Ad), herpesvirus, adeno-associated virus (AAV), and SV40 to the CNS [87,88,89]. In an adult mouse model of mucopolysaccharidosis type IIIB, this method of treating neurodegenerative illness was shown to be therapeutically helpful (MPS IIIB) [90].

The syndrome is characterized by substantial CNS neurodegeneration owing to lysosome accumulation of heparan sulfate and its derivatives, which is caused by N-acetylglucosaminidase (NAGLU) alterations. However, MPS IIIB has major somatic symptoms such as hepatosplenomegaly and skeletal abnormalities; once enough vector can penetrate the CNS and lysosomal storage in the peripheral nervous system, it is a suitable candidate for systemic gene transfer [91]. AAV serotype 2 (AAV2), which does not normally traverse the BBB, and mannitol pretreatment were used to deliver the faulty NAGLU gene intravenously. While the number of brain cells transduced was small (about 1–2%), the homogenous dispersion throughout the brain parenchyma, along with the cross-correction effects of the secreted NAGLU enzyme, allowed for extensive CNS disease correction. The timing of IV vector delivery in connection to mannitol administration was critical, with 8 min after pre-treatment generating 10-fold greater transduction than 5 or 10 min [90].

A highly concentrated and targeted vector transduction pathway can be achieved by transiently rupturing the BBB using magnetic-resonance-guided focused ultrasound plus IV-administered microbubbles, followed by IV-administered AAV vector [79]. This BBB permeabilization technique was created to treat a wide range of illnesses, and it depends on the mechanical action of lipid/gas microbubbles in reaction to ultrasonic pressure pulses to damage the brain endothelium for 6 h [92].

The new work recommends that the whole brain be permeabilized for gene therapy in a variety of disorders. Following IV treatment and FUS sonication in a grid scan method, a self-complementary AAV9-CMV-GFP vector rapidly and efficiently penetrated the whole brain of mice. When compared to non-FUS-GFP-injected mice, nearly the entirety of extracts of FUS-treated GFP-injected mice exhibited a rise in vector DNA (19.8 times), GFP mRNA (16.4 times), and GFP protein levels (17.4 times). There had been a 7.3-fold increase in infected cells in the cortex, hippocampus, and striatum, in case of an increase in GFP levels. In the brains of rats treated, no harmful consequences were seen. The use of FUS and AAV-based gene transfer to treat neurological genetic diseases is a significant step forward [93].

Despite paracellular transport over a compromised BBB showing promise in animal models, the alternative method of crossing the BBB through receptor-mediated transcytosis has recently shown greater promise. These methods are divided into three categories: employing a viral vector that naturally crosses the BBB (usually, AAV9 [94]); altering vectors to attach to a particular transcellular transport receptor; and changing vectors to attach to a specific transcellular transport receptor [95]. Guided evolution was used to generate a vector that binds with an appropriate receptor despite specifying which one. To carry huge molecules across the BBB, they all require highly specialized channels, typically targeting well-studied transporters like the transferrin receptor, LDL receptor-related proteins (LRP-1 and LRP-2), or insulin receptor [96].

#### 3.2.3. Exosome-Mediated Transport across the Blood–Brain Barrier

Exosomes are naturally occurring vesicles found in virtually every human body cell. As a result, there has been considerable progress in understanding exosomes, including their function, processes, and compositions, over the last two decades. Extracellular vesicles, as endogenous NPs with the capability to bridge BBs, are being hailed as possible forerunners of a new era in medicine, with a variety of applications including prevention, intervention, and pharmacological and gene therapies [97]. As more information becomes available, it will be possible to alter EVs to become specific medication delivery systems (DDSs). The administration methods, payload, structure, selectivity, and reception for bridging BBs, on the other hand, are unclear and require further research [98].

To that purpose, various nano- and micro-sized structures, such as EVs [99], are classified according to their cellular origin, dimensions, biogenesis, and physicochemical features [100]. According to popular scientific assumptions, EVs are formed via the plasma membrane and endosomal processes. Exosomes, as previously said, are regarded as one of the most significant types of EVs, having an endosomal origin and a diameter of 50–100 nm [101] in cellular homeostasis, intracellular interaction, and molecular pathways that are engaged in physiological and pathological functions [102]. Donor cells can affect specific signaling pathways in recipient cells by transferring cytoplasmic proteins and lipids, as well as genetic components like iRNA, which are involved in diverse signal transduction pathways [103].

The key location in the BBB that governs exosomal transport is the EC. When circulatory exosomes come into physical contact with BBB ECs, various general processes involved in EV absorption, including the speeding-up of the influx of exosomes from the blood into the brain tissue, endocytosis, micropinocytosis, phagocytosis, and plasma membrane fusion are used [104].

Exosomes are recognized to have the ability to cross the BBB under normal circumstances. It should not be overlooked that the development of inflammatory circumstances might disrupt the BBB, resulting in increased exosome transfer and permeability. A problem that supports this notion is the rapid movement of exosomes from the CSF to the bloodstream following the onset of CNS inflammatory alterations [105].

Because of broad capillary networks and specific subsets of immune cells with phagocytic receptors, a considerable percentage of systemically injected exosomes is soon caught in the hepatic, lung, and splenic tissues, according to several studies [74]. Through several investigations, unaltered exosomes may easily disseminate in biofluids via free dispersion without targeting capabilities [106]. As a result, surface modification tactics can be used to alter exosome targeting capabilities [107]. Conjugation of specific ligands to the surface of transplanted exosomes may improve the interaction with specific moieties on cells, while adding labeled radioactive, fluorescent, and MRI agents at the same time is a useful approach to track in vivo. In line with these descriptions, surface modification has been proposed as a potential method for increasing exosome trafficking via the BBB [108]. Exosome delivery has been accomplished via a variety of modification ways so far. Surface treatment using ap-tamers, non-covalent and covalent alterations, multivalent electrostatic interactions, and genetic engineering have all been utilized to alter the exosomal surface [109].

Exosome surfaces are functionalized with tiny and large biomolecules and polymers in click chemistry, for example, without altering exosome activity [108]. Chemical alteration is a more appealing modality due to its simplicity of synthesis, high throughput, and the large number of chemical reactions accessible [108,110,111]. The use of hazardous solvents like dimethyl sulfoxide, temperature and pressure fluctuations, and osmotic changes that might harm exosome structure are all inevitable drawbacks of traditional chemistries [109].

The use of a natural receptor on the luminal surface is a sensible alternative for increasing exosome transcytosis rates. LDLRs are membrane-bound receptors with a wide range of actions based on this. Endothelial endocytosis is mediated by these receptors, which have the potential to connect to a variety of ligands in addition to lipoprotein metabolism [110]. Exosomes containing KLA suppressed the development of human tumoroid U87 cells in vitro and increased extravasation via the BBB, leading to longer median survival rates than exosomes without KLA [112]. According to single-cell investigations, LDLR subsets also contribute to the route of endocytic cargo to the lysosomes. However, there is no clear evidence that LDLRs are involved in endocytosis or transcytosis [113]. Some data suggest that the LDLR2 subgroup, specifically, is involved in transcytosis [114]. According to these assertions, the formation of KLA-loaded exosomes should prioritize the activity of LDLR subsets that facilitate endothelial transcytosis over intracellular metabolism and protease activity. Otherwise, biotherapeutic compounds would be eliminated at the BBB level before reaching their target location.

Exosomes might be thought of as clever carriers for medication delivery to the brain parenchyma, according to earlier research and the mechanisms discussed in this article. Researchers may be able to improve the exosome–ECs interaction on the surface of the BBB and facilitate exosome passage, thereby increasing the efficacy of drug delivery to the abluminal side of the BBB, if they have a better understanding of the recruited mechanisms of exosomes passing through BBB and related challenges.

#### 3.2.4. Intranasal Route of Delivery

Intranasal (IN) injection of medicines to the central nervous system has a number of advantages in the treatment of neurologic diseases. The blood–brain barrier restricts the use of most therapeutic drugs designed to treat memory loss and neurodegeneration because it restricts CNS penetration, based on drug size or charge [115]. Although invasive delivery techniques (such as intracerebroventricular) were used to circumvent the BBB, these procedures are not feasible for use in humans due to a variety of factors, comprising accessibility, safety, and cost. IN administration crosses the BBB and offers a non-invasive approach to invasive medication administration by providing medications directly into the CNS from the nasal cavity. Many CNS medications, even those that may cross the BBB when taken systemically, benefit from non-invasive IN administration because it targets therapy to the CNS, reducing systemic exposure and side effects. IN delivery does not necessitate changes to CNS treatments, and therapeutic delivery to the CNS is quick, taking only minutes [116]. Frey invented the IN delivery technique in 1989 [117] to transfer neurotrophic factors to the CNS (such as nerve growth factor [NGF] and fibroblast growth factor-2).

Therapeutics delivered intranasally enter the CNS through the olfactory and trigeminal neuronal pathways. The nasal cavity is innervated by both the olfactory and trigeminal nerves, directly linking to the CNS. The olfactory pathway was once thought to be responsible for direct medicinal delivery from the nose to the brain [117,118]. The trigeminal pathway’s role in IN distribution to the CNS, particularly to caudal brain areas and the spinal cord, has recently been acknowledged [119,120].

IN delivery of neurotrophins appears to be a non-invasive strategy to direct neurotrophins to the CNS to treat neurodegeneration, according to growing data. IN given NGF decreased neurodegeneration and enhanced cognitive function in a mouse model of Alzheimer’s disease [121,122]. IN IGF-I decreased brain damage and neurologic impairments in a rat stroke model [123]. In addition, after IN injection of FGF-2 to adult mice, brain neurogenesis was promoted in the subventricular zone. In animal models of Alzheimer’s disease, neurodegeneration, tau pathologies, amyloid accumulation, and loss of memory have all been shown to be reduced by IN-activity-based neurotrophic factor and its functional peptide fragment NAP [124]. IN NAP is currently being investigated in phase II clinical studies as a potential Alzheimer’s disease and mild mental impairment treatment.

IN delivery is a non-invasive technique of delivering medications to the CNS, bypassing the BBB, and limiting systemic exposure and negative effects. This innovative approach has previously been shown to boost memory in healthy persons and Alzheimer’s disease sufferers. IN delivery has the potential to change the way Alzheimer’s disease and other neurodegenerative illnesses are treated.

#### 3.2.5. Modulating Blood–Brain Barrier Permeability

There are four different types of adenosine receptors. A2A and A2B activate adenylate cyclase by binding to the Gs protein, whereas A1 and A3 decrease adenylate cyclase activity by binding to the Gi/0 proteins [125]. The subunit determines the affinity of A2A receptors for Gs proteins; the greatest contact occurs with G proteins that include the four subunits. Stimulation of the receptors leads to a rise in cAMP amount in the cell. The expression of the A2A receptor is regulated by protein kinase C, which is altered in pathological circumstances. Using radiographic approaches, A2A receptors were discovered on leukocytes (e.g., neutrophils), platelets, blood vessels, and even inside the CNS, such as in the striatum [126]. Adenosine, as well as inosine and other exogenous compounds utilized in treatment, activate these receptors [127,128]. The expression of proteins that regulate BBB function may be changed in diseased situations. In the context of hypoxia or inflammation, substances like interleukin- or tumor-necrosis factor boost the expression of the A2A receptor in glial cells [129].

The activation of A2A receptors reduces platelet aggregation and controls blood pressure by vasodilation, according to Ledent et al. A2AR agonists influence pain pathways [130]. A2A receptor agonists have been shown to have a vasodilating impact in the coronary arteries of rats and the mesentery of dogs. Gong Zhao et al. discovered that the CVT-3146 adenosine receptor agonist’s vascular resistance-lowering impact was dosage-dependent and had a more powerful effect on coronary arteries than adenosine [131].

Another essential role of A2A receptor agonists is to prevent tissue from injury during ischemia-reperfusion, such as in hemorrhagic shock, by lowering inflammation (affecting neutrophils, platelets, macrophages, and T cells) [132,133]. Furthermore, adenosine agonists in the central nervous system (CNS) reduce inflammation and protect nerve cells [134].

#### 3.2.6. Focused Ultrasound for Brain Diseases

In recent years, ultrasound has become a popular method for allowing medications to pass across the BBB. MEUS, or microbubble-enhanced diagnostic ultrasonography, is a non-invasive approach that helps medications pass the BBTB by enhancing BBTB permeability in glioma patients. Major proteins in TJs in the BBB include claudins, occludin, and JAMs [135]. Ultrasound irradiation and microbubbles might suppress the expression of these TJ proteins [135], opening the BBB over a short period of time without causing injury to normal brain tissue [136]. Furthermore, MEUS increased the expression of KCa channels in glioma cells, according to Ningaraj et al., promoting pinocytosis and, as a result, increasing BBTB permeability. The BBB, in addition to the BBTB, is still a barrier to medication delivery in brain tumors. When focused ultrasound (FUS) is paired with microbubbles, the permeability of the BBTB in brain tumors can be increased while the BBB in the adjacent tissue is disrupted [137].

**Figure 4:**
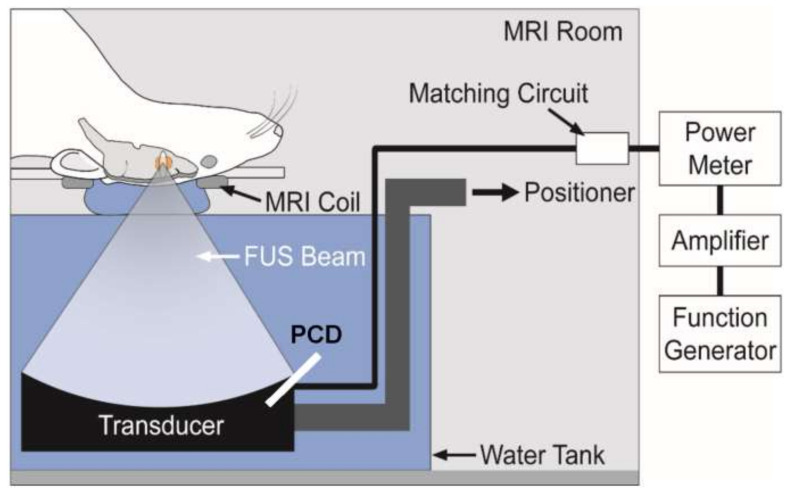
Schematic representation of the MRI-guided FUS system used by Park et al. Reprinted from Ref. [138], PLOS, 2017.

Park et al. exploited DCE-MRI to investigate the administration of doxorubicin using a combination of FUS and microbubbles. To transport doxorubicin to a rat brain cancer and the normal brain, researchers used FUS and microbubbles. They showed that using both techniques boosted drug retention duration in the tissue over 24 h while also improving drug crossing of the BBB and BBTB. Furthermore, MEUS’ ability to temporally decrease P-gp expression is intriguing. P-gp was suppressed for up to 48 h and then recovered after 72 h using MEUS, and the amount of induced suppression could be varied by changing instrument parameters. Non-human primates were given FUS at various sonic pressures to study the physiological changes in the brain caused by the BBB opening caused by FUS [138] (Figure 4).

In addition, Dallan McMahon et al. noted in their clinical study that, following FUS+MB exposure, DEX treatment was observed to speed up the repair of BBB integrity in the targeted dorsal hippocampus while also preventing an increase in the generation of inflammatory markers. These findings imply that DEX might be used to control the amount to which BBB permeability is enhanced, allowing for repeated FUS+MB exposures with a lower risk of tissue harm caused by cumulative negative effects. The outcomes reported here indicate that DEX administration following FUS+MB exposure may be warranted in clinical cases where vascular damage is suspected and the goal of treatment is to restore or preserve neural function, based on its widespread clinical use and well-documented mechanisms of action [139].

The pharmacokinetic research indicated that FUS opened the BBB locally and transiently, facilitating medication transport. The amount of BBB opening, and hence the drug concentration in the brain, was influenced by brain inhomogeneity and acoustic pressure. The fundamental concepts of ultrasound-mediated medication administration may be discovered, as well as a full description of the possibilities [140]. In addition to FUS, medication distribution across the BBB was aided by transcranial magnetic stimulation (TMS), which promotes neuronal activity and boosts glutamate release [141]. TMS improved BBB permeability in 10 of 15 patients with malignant brain tumors in preliminary clinical research, showing that TMS might be used in clinical settings to improve medication delivery into the brain [142].

Roland Beisteiner et al. describes a clinical sonication approach based on single ultrashort ultrasound pulses (transcranial pulse stimulation, TPS). A first clinical trial employing ultrasonic brain stimulation is also described, as well as the first findings of long-term effects. Large safety margins and dose-dependent neuromodulation are seen in preclinical data. Patient examinations suggest that the medication is well-tolerated and that there are no serious adverse effects. TPS therapy improves neuropsychological scores dramatically, with improvements lasting up to three months and correlating with an upregulation of the memory network (fMRI data). The findings support a broad neuroscientific use of the approach, as well as its translation into therapeutic treatment and randomized sham-controlled clinical trials [143].

There are several potential advantages of concentrated US (quality of life, lifespan, lower expenses, shorter treatment duration), but are there any disadvantages? Therapy for massive brain tumors, for example, may take a long period, but there is now no other treatment option [144].

#### 3.2.7. Nanoparticles for Brain Imaging/Diagnostics

The use of nanoparticles in tumor imaging and diagnosis has received much attention. However, due to the challenges posed by the BBB, little research has been conducted on brain imaging for CNS illnesses. The advancement of imaging technology, particularly MRI and computed tomography (CT), has revolutionized neurodegenerative disease care and prognosis. BBB disruption may be measured quantitatively in ischemic stroke patients using DCE-MRI [145]. The dynamic course of the damage and BBB breakdown following intracerebral hemorrhage, which is a substantial source of morbidity and mortality, has also been tracked using multimodal MRI [146]. Quantitative and visual measurement of increasing BBB permeability may assist in identifying suitable treatment measures beyond the stated time frame, in addition to diagnostics and monitoring therapeutic outcomes. The first therapy for acute stroke patients is tPA. However, tPA has the potential to cause bleeding. Hemorrhage transformation was linked to increased BBB permeability in CT angiography studies [147]. Before tPA therapy, CT might be used to determine the risk of bleeding. Gd-micelles, which were created as an MRI contrast agent for tumor imaging, were also employed in a rat model to investigate BBB permeability [26]. The ischemic hemisphere had a substantial contrast in the MRI scans, showing macromolecule permeability via the BBB. The Gd-micelles stayed in the ischemic hemisphere because of their enormous molecular weight. As a result, the Gd-micelles MRI contrast agent could visualize BBB opening to quantify bleeding risk.

These findings show that CT and MRI brain imaging might be used to screen people for brain disorders. Patients are exposed to a significant amount of radiation with the current CT procedure. Nanoparticles have been produced to improve tumor diagnosis using CT or MRI. As the BBB leaks under specific illness situations, tumor diagnostic nanoparticles, such as Gd-micelles, can be used to diagnose brain disorders. Advanced imageable nanoparticles might potentially reduce the amount of contrast chemicals used in CT and MRI procedures, making them safer.

Nanosized imaging agents and nanocarriers functionalized with imaging agents are the two main types of nanoparticles used in brain imaging. Furthermore, by functionalization with therapeutic drugs, they can be created as theranostic agents [148]. Thus, by using theranostic nanoparticles in disease management, the disadvantages of traditional nanoparticles, such as patient compliance and safety, might be solved [149]. Iron oxide, gold, and manganese oxide are all commonly researched imaging agents.

Because of their unusual physicochemical and superparamagnetic features, iron-oxide-based nanoparticles have attracted a lot of interest as contrast agents. Thus, the utilization of iron-oxide magnetic nanoparticles might enhance medical applications such as cell labeling and sorting, cell transfection, and diagnostic imaging based on MRI, PET, or multimodal imaging [150]. In vitro investigations have shown that theranostic iron oxide nanoparticle surface modification with caffeic acid can be used for glioblastoma MRI and reactive oxygen species formation as a treatment method [151]. Furthermore, iron oxide nanoparticles functionalized with phosphonate polyethylene glycol chains and covalently connected to cyclic RGD were used in vivo for MRI of glioblastoma in mice [152].

As contrast agents for preoperative, intraoperative, and postoperative imaging, gold nanoparticles have piqued the scientific imagination [153]. Furthermore, by adding chemical compounds and targeting molecules to the surface, gold nanoparticles may be employed as multifunctional contrast agents with extended circulation duration, allowing for larger imaging windows [154]. The creation of a target-specific imaging system based on peptide-coated gold nanoparticles for identifying glioma cell biomarkers has validated the imaging agent’s fluorescence signal-based feature [155]. Furthermore, gold nanoparticles may allow for the imaging of implanted stem cells within the brain. One study proposed using gadolinium-labeled DNA gold nanoparticles for MRI tracking of brain stem cells [156].

As gadolinium is highly toxic and is present in all commercially available intravenous agents, researchers have been concentrating their efforts on producing gadolinium-free contrast agents [157,158]. In their study, Eric M. Gale and Peter Caravan reported that manganese is the most promising replacement for gadolinium [158]; manganese oxide nanoparticles were used for in vivo glioblastoma MRI [159].

#### 3.2.8. Liposome-Based Strategies for Effective Drug Delivery across the Blood–Brain Barrier

Liposomes have been studied extensively for their use in drug administration and in vivo bioimaging for the treatment and/or diagnosis of neurological illnesses such as Alzheimer’s, Parkinson’s, stroke, and glioma due to their unique physicochemical properties [160].

Liposomes can include hydrophilic, lipophilic, and hydrophobic medicinal substances due to their distinct physicochemical properties. Hydrophilic chemicals can be found at the interface between the lipid bilayer and the exterior water phase, or they can be entrapped in the watery core of liposomes. Lipophilic or hydrophobic medications are virtually fully captured in the liposomes’ hydrophobic core of lipid bilayers. The usage of cationic lipids also allows for the adsorption of polyanions like DNA and RNA [54,55].

They also exhibit strong biocompatibility and biodegradability, as well as minimal toxicity, drug-targeted delivery, and controlled drug release. The liposome surface can be further changed by adding macromolecules such polymers, polysaccharides, peptides, antibodies, or aptamers to increase blood circulation and brain-specific delivery [54]. Unfortunately, liposomes are not currently used in clinical practice to deliver brain-specific drugs [161,162]. Several, on the other hand, are either licensed for clinical use or are undergoing clinical trials [163,164].

Intranasal administration of rivastigmine [165] or galantamine liposomes [166] was already established to be a practical and effective method of increasing medication bioavailability in the brain. Intranasal administration of liposomes containing quercetin, an antioxidant, was also found to be a viable method of delivery [167]. The usage of quercetin liposomes has been found to reduce neuronal oxidative stress, which is a key factor in the neuropathogenesis of Alzheimer’s disease [168]. 

Currently existing treatments for Parkinson’s disease are symptom-focused and have little effect on disease progression [169]. Levodopa, often known as L-dopa, is a natural precursor to dopamine that has been utilized in the clinic for several years. Levodopa, on the other hand, cannot be given alone because it is transformed to dopamine by the peripheral dopamine-decarboxylase enzyme, which produces tiredness, nausea, and dyskinesia [170]. This challenge was solved in a latest study that developed liposomes for site-specific transport of levodopa into the CNS [160,171]. Levodopa encapsulated in chlorotoxin-modified stealth liposomes demonstrated to be an effective nanocarrier, raising levodopa content in the substantia nigra and striatum. In a similar manner, intraperitoneal injection of liposome formulations containing anti-PD medicines was found to increase dopamine release in the striatum area [172].

Drug delivery methods for the brain have been identified and developed. Since numerous targeting techniques have been published demonstrating abilities to enter the brain and target the tumor, liposomes have been described as a potentially helpful approach for achieving greater therapeutic effects in the treatment of gliomas.

To overcome the disadvantages of RES (resveratrol) as a free medication, it was placed into PEGylated liposomes (RES–L). The liposome surface was modified with transferrin moieties (Tf–RES–L) to make them cancer-cell-selective since transferrin receptors (TfRs) are up-regulated in GBM. The goal of the liposomal nanomedicines produced in this study was to improve the physico-chemical characteristics of RES and to use the passive and active targeting capabilities of liposomes to treat GBM efficiently. The RES–Ls were stable, had a high drug-loading capacity, had a long in vitro drug-release time, and were simple to scale up [172].

**Figure 5:**
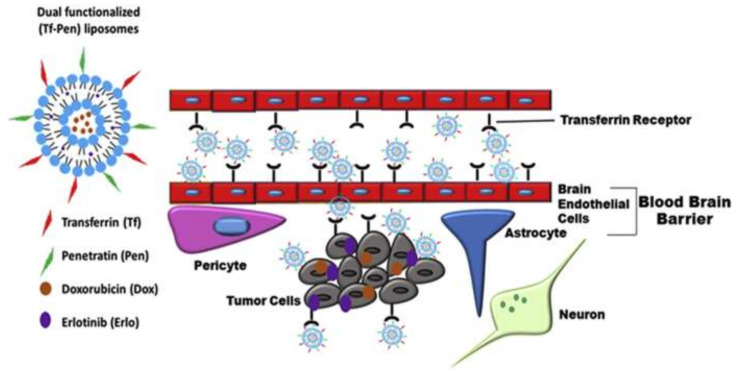
Schematic representation of dual-functionalized (Tf–Pen) liposomes translocation across the BBB, followed by endocytosis into glioblastoma cells. Reprinted with permission from Ref. [173], Copyright 2019, Elsevier.

In another study, researchers created a dual-functionalized liposomal delivery system containing transferrin (Tf) for receptor-mediated transcytosis and a cell-penetrating peptide–penetratin (Pen) for increased cell penetration in another investigation (Figure 5). They put doxorubicin and erlotinib inside liposomes to help them go through the BBB and into the glioblastoma tumor. In vitro cytotoxicity and hemocompatibility testing revealed good biocompatibility for in vivo use [173].

#### 3.2.9. Solid-Lipid Nanoparticles

SLNs are lipid-based biocompatible nanocarrier systems made up mostly of lipid or modified lipid nanostructures (triglycerides, fatty acids, or waxes) with diameters ranging from 10 to 1000 nm [174]. Both hydrophilic and lipophilic medicines can be distributed in the solid hydrophobic lipid core of SLNs. They play an important function in the BBB’s reticuloendothelial system (RES) crossing [175]. Due to its solid lipid composition rather than being an aqueous solution, this colloidal nano-carrier was created as a superior replacement for polymeric nanoparticles and liposomes to protect active-drug-counteracting biochemical degradation [174].

Recent studies using SLNs as a carrier system have tried to carry medications over the BBB to the brain. SLNs are a new smart drug delivery system with innovative features for the treatment of neurological disorders, with desirable properties such as nanodiameter range, site-specific targeted delivery (via receptor-mediated transcytosis across brain capillary endothelial cells), physical stability, ability to escape the reticulo-endothelial system, extended blood circulation time, sustained release, and nontoxic, biodegradable, and biocompatible qualities [176].

During intravenous delivery, drug-loaded SLN carriers have a higher accumulation and targeting capability in the brain than in other organs, according to research [177]. SLNs can be given to neuronal locations by oral, inhalational, or parenteral methods as novel delivery vehicles encapsulating active medicines for the treatment of CNS diseases [178]. Following that, SLNs repair neuropathologies by interfering with abnormal signaling pathways as well as metabolism [179]. SLNs loaded with medicines have a number of potential uses in the treatment of CNS illnesses.

They can also distribute the active drug ingredients in a target-specific and regulated way, with fewer potential toxicity risks, because to their unique physicochemical character. Furthermore, SLNs have therapeutic benefits for successful brain medication delivery, including decreased side effects, longer drug half-life, and the potential to improve drug capacity to penetrate the BBB. Despite this, SLN has certain drawbacks, including a smaller drug payload, a complicated physical state of the lipid content, and storage and administration stability issues (gelation, increase in particle size, drug expulsion) [55]. Given the existing disadvantages of SLNs, further research and development is required to make them an optimal CNS drug delivery strategy for treating the widest range of neurological illnesses.

Many research studies on the use of drug-loaded SLNs for the treatment of various CNS disorders, including Alzheimer’s disease, Parkinson’s disease, multiple sclerosis, brain tumors and cancer, epilepsy, ischemic stroke, and certain neurodegenerative disorders, have been published in recent years and are still ongoing [180].

### 3.3. Mechanisms by Which Nanosystems Pass through the BBB

Nanoparticles are limited in their ability to penetrate the BBB and, as a result, get access to the brain. Nonetheless, several ways for crossing the BBB by nanoparticles have been tackled, taking advantage of the various physiological factors underpinning this process. The attempted delivery methods include using receptor-mediated endocytosis, transcytosis, or transporters, yet they have not been proved completely efficient for exploiting the full therapeutic and diagnostic potential of nanoparticles [181]. Thus, BBB permeability was sought to be enhanced through the employment of focused ultrasound in synergy with the administration of microbubbles or via different nanoparticles’ surface modification possibilities (e.g., coatings, targeting ligands). 

The primary ways that have been reported as promising for nanoparticles permeating the BBB are presented in Table 2.

**Figure 6:**
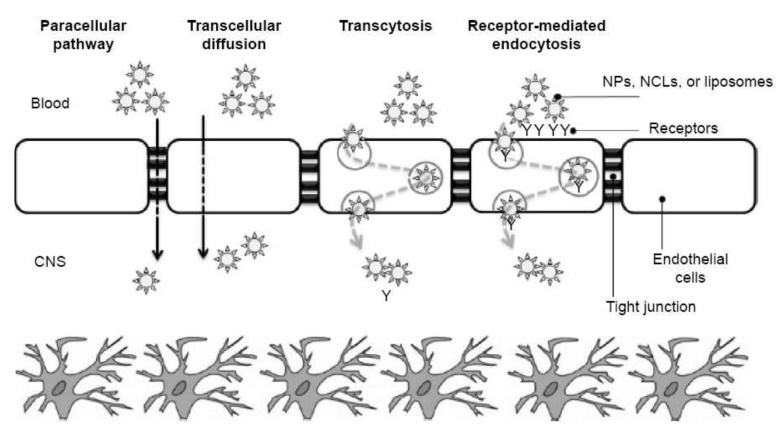
Main BBB crossing pathways for nanosystems. Abbreviations: CNS–central nervous system; NCL–nanostructured lipid carriers; NPs–nanoparticles. Reprinted from Ref. [188], Dove Press Ltd., 2015.

For a better understanding, the main mechanisms for BBB crossing by nanosystems are visually represented in Figure 6.

## 4. In Vitro and In Vivo Models of BBB for Assessing Drug Delivery

In light of our current understanding of the BBB’s operation, it is reasonable to state that in vivo models are the most well-known and oldest models for simulating the BBB’s structural and functional complexity [189]. The utilization of a living brain in its natural environment is the key benefit. However, adopting in vivo technologies as a high to medium throughput screening procedure is challenging and expensive, hence they are only used in the early phases of drug development [190]. When it comes to the sheer quantity of samples that can be analyzed in a given length of time, the scientific world has finally realized the value of in vitro investigations.

Despite the fact that almost half of all chemicals examined cannot be translated into people, in vitro models are still less expensive and quicker than in vivo studies [191]. A good BBB model should be repeatable, have a realistic architecture, functional expression of transporters, and be simple to set up [189].

In vitro models have been adopted for specific research despite the fact that no known in vitro model can reproduce the NVU of an in vivo system. In general, the goal of in vitro models is to create a highly malleable and controllable environment that allows us to create a system that is slightly similar to a living system in order to assess pathophysiological responses to external stimuli that would otherwise be too difficult to reproduce and characterize in vivo at an early stage of drug development [189]. Over the years, several models have been constructed to investigate the role of BBB in drug delivery; these may be divided into non-cell-based and cell-based in vitro models.

## 5. Clinical Trials and Products Available on the Market

With a rate of 19.6%, central nervous system trials have increased the most since the beginning of 2021 [192]. Being a topic of high research interest and clinical applicability, it is no surprise that numerous studies concerning BBB have reached the stage of clinical trials. The scientific community has focused on investigating novel methods for enhancing BBB permeability for drug delivery and imaging, bypassing BBB or BBTB in various neurologic conditions, and evaluating the safety of repeated BBB opening (Table 3).

Despite the abundance of recent clinical studies in the field, the market is still scarce in products for enhancing BBB permeability for efficient drug delivery. Nonetheless, several patents have been reported. For instance, Armagen Technology Inc. holds a patent for “Agents for blood–brain barrier delivery” (CA2661042C). Their invention offers compositions, methods, and kits for augmenting transport across the BBB, while allowing agent activity to remain substantially intact once reaching the target. These therapeutic, diagnostic, or research agents (i.e., antibody composition comprising a pharmaceutically acceptable carrier and a fusion antibody) are transported across the BBB via one or more endogenous receptor-mediated transport systems. 

The same biotechnology company holds another patent entitled “Fusion proteins for blood–brain barrier delivery” (CA2625293C). This newer invention consists of a composition made of an agent covalently linked at its amino terminus to an antibody that crosses the BBB through an endogenous BBB receptor selected from the group consisting of the insulin receptor, transferrin receptor, leptin receptor, lipoprotein receptor, and the IGF receptor. The reported pharmaceutical composition produces an average elevation of concentration of the agent in the brain of at least 5 ng/g brain following peripheral administration, while the agent binds to a receptor and induces neuroprotection. 

Armagen has proposed a similar active patent in Japan under the name “Fusion protein for blood–brain barrier transport” (JP628371B2). This invention represents a composition comprising an agent (i.e., erythropoietin) covalently bound by a monoclonal antibody (HIRMAb) and the amino terminus to the human insulin receptor. As in the case of the previously mentioned patent, this formulation also allows an increase in the intracerebral concentration by at least 5 ng/g after peripheral administration while ensuring a neuroprotective effect. 

In addition, it can be expected that undergoing clinical trials may soon offer promising results, leading to the entrance into the market and/or clinical practice of novel performant devices, procedures, and drug formulations for enhancing BBB permeability without compromising patients’ safety. 

## 6. Perspectives and Future Research Directions

Pre-clinical studies have revealed focused ultrasound as a viable therapeutic approach for recurring, non-invasive, and transient BBB opening for the therapy of CNS disorders, based on significant proof-of-concept in multiple animal species. The efficacy of ultrasound-mediated BBB permeability for the controlled and regulated transportation of drugs, such as antibodies, to the brain, as well as larger vehicles for drug delivery applications, such as liposomes, polymeric nanoparticles, and even viruses, was also shown in these studies. This opens the door to using this modality to deliver potential new treatments such as checkpoint blockade antibodies for extracranial tumors, modulating protein expression using transcription factor genes, micro-RNAs, and shRNAs to target immune signaling in diseased tissue, and repurposing drugs that have performed poorly, for example, because they did not reach their goal in therapeutic quantities.

For optimal BBB rupture, a multitude of clinical studies have demonstrated appropriate parameters for ultrasound parameters such acoustic frequency, center frequency, pulse repetition frequency, ultrasound pulse duration, and microbubble injection. However, the majority of these investigations have been carried out on tiny animals. Until this modality can be effectively integrated to larger animals and, sooner or later, to humans, a multitude of technical difficulties must be addressed, including the fact that the heterogeneity of the skull can induce ultrasound beam disturbance and pressure attenuation, the need for standardized ultrasound techniques and trying to report, and quantitative metrics for establishing BBB opening. Furthermore, current microbubbles have not been specifically designed for BBB opening using ultrasound.

This emphasizes the need for creating and optimizing standardized microbubbles. In order to deliver tailored therapy while reducing adverse effects, translational investigations are necessary for enhanced real-time monitoring of the effects of ultrasound on the BBB. Aside from these physical constraints, there are a lot of biological unknown factors, such as the brain’s heterogeneity and the NVU’s cellular components’ responses to ultrasound. Cellular NVU approaches relying on patient-derived ECs should aid in better understanding the BBB’s molecular impacts. In addition, research employing new and improved BBB models will give information on cellular interconnections and signals in both healthy and diseased conditions.

More study is needed to better understand how skull and brain characteristics, as well as microbubble properties, influence BBB permeability and how they alter with aging and disorder, as well as the ability of ultrasound in enhanced cellular and animal models of brain diseases.

Liposomes, nanoparticles, and dendrimers have shown promising results following technological advancements. Alternative routes beyond the BBB have also showed great commercial promise. Furthermore, in order to achieve a beneficial brain-targeted drug-delivery system, some fundamental concerns must be addressed in the near future, such as (1) factors affecting the system’s in vivo performance should be well-explained and evaluated (for example, in in vivo studies describing the treatment of glioblastoma, the BBB in tumors is less tight than in healthy brain tissue; this may influence the study’s outcome), (2) more focus should be put on the development of novel drug delivery systems, (3) targeting the efficiency of such systems before clinical application requires significant improvement; (4) hydrophobic drugs may leak from the delivery system and then enter the brain (and thus, proof of drug penetration into the brain does not (necessarily) imply proof of delivery system penetration); and (5) a uniform method of preparation for the development of a nanoparticulate system should be developed to achieve a more homogeneous system. The key to developing a successful delivery system is to consider not just effective brain targeting formulations, but also less toxic and safe medications in order to provide patients with a better and healthier future.

## 7. Conclusions 

This review has analyzed current medicine delivery techniques to the brain in the last five years. A thorough understanding of BBB disruption is required to build successful medication delivery methods for brain illnesses. Recent developments in research have shown the permeable BBB in brain damage and the processes of BBB control. In addition to well-established technologies like viral vectors and nanoparticles, novel non-invasive techniques like MEUS and TMS have just been developed to temporarily open the BBB and increase medicine absorption in the brain. It is reasonable to suppose that new delivery systems will help in the diagnosis of brain illnesses. Along with a better understanding of the leaky BBB, previously developed nanoparticles targeting tumors via the EPR effect could be exploited to treat brain diseases. Gliomas come in a wide variety of sizes and shapes, with typical permeability at the perimeter. As a result, a combination of approaches for accessing both permeable and non-permeable BBBs may be necessary.

Furthermore, further studies are needed on the dynamics of BBB disruption, which will aid in the design of adequate delivery systems that take advantage of the leaky BBB. Another crucial topic that warrants additional research is the effect of aging on BBB dysfunctions. Brain medication delivery systems that have considered the effects of aging and have been evaluated in animals of various ages are uncommon in the literature. In conclusion, the intricacy of the BBB necessitates more extensive investigations on delivery tactics. Still, it may also provide a unique opportunity to build effective delivery systems to treat diverse brain illnesses.

## Figures and Tables

**Table 1:** Strategies for brain drug delivery.

Type of Procedures	Strategies	Advantages	Limitations	References
Invasive approaches	Blood–brain barrier transient disruption	Transient opening; can achieve therapeutic concentrations	Non-targeted, entire BBB is disrupted	[47,48]
	Intracerebroventricular and intrathecal infusion	High gene transfection efficiency	Safety concerns; direct brain injection; crossing the BBB; high dose by intravenous administration	[47,49,50]
Non-invasive approaches	Chemical modification of drugs	Option for personalized medicine	Difficult to achieve therapeutic concentration in vivo; expensive and difficult to develop; non-targeted	[48]
	Virus-mediated blood–brain barrier delivery	High gene transfection efficiency	Safety concerns; direct brain injection; crossing the BBB; high dose by intravenous administration	[47,49,50]
	Exosome-mediated blood–brain barrier delivery	Gene delivery to the brain; potential ability to cross the BBB	Exosome donor cells; loading procedure; in-vivo toxicity and pharmacokinetics	[47]
	Intranasal route of delivery	Bypass the BBB through nasal administration	Suitable for low dose	[51]
	Modulating blood–brain barrier permeability	Transiently open the BBB	Mismatch between findings in rodents and humans	[52,53]
	Focused ultrasound for brain diseases	Therapeutic concentrations; targeted to sub-millimeter regions in the brain	Only relatively short-term studies have been performed	[48]
	Nanoparticles for brain imaging/diagnostics	Enhance imaging; cross the BBB through the hyper-permeable BBB under disease conditions	Cross the BBB; understand dynamic changes in the BBB	[22]
	Liposome-based strategies	Exhibit strong biocompatibility and biodegradability; minimal toxicity; drug-targeted delivery; controlled drug release	Liposomes are not currently used in clinical practice to deliver brain-specific drugs	[54]
	Solid-lipid nanoparticles	Site-specific targeted delivery (via receptor-mediated transcytosis across brain capillary endothelial cells); physical stability; ability to escape the reticulo-endothelial system; extended blood circulation time; sustained release; nontoxic; biodegradable; biocompatible	Smaller drug payload; a complicated physical state of the lipid content; storage and administration stability issues	[55]

**Table 2:** Approaches for improving BBB crossing of nanoparticles.

Primary Method	Sub-Method	Description	References
Paracellular pathway	-	Ultrasound/microbubbles and osmotic pressure are two methods for disrupting tight junctions between neighboring endothelial cells, both of which increase BBB permeability locally, allowing nanoparticle entrance. However, when the BBB’s homeostatic role is lost, this strategy carries certain hazards, as it would enable uncontrolled entrance of various substances into the brain, potentially resulting in cerebral toxicity.	[182]
Transcellular diffusion	-	The simplest transcellular route involves passive diffusion through the cell membrane and cytoplasm. To cross the phospholipidic bilayer of the membrane, nanoparticles must be small enough and at least partially lipophilic.	[183]
Transcytosis pathway	Adsorptive transcytosis	The surface characteristics of the nanoparticles make it easier for the nanoparticle and its payload to attach to the endothelial cells’ luminal plasma membrane. Because the plasma membrane of endothelial cells is negatively charged, positively charged nanoparticles are more likely to undergo this process than neutral or negatively charged ones. Nanoparticles coated with wheat germ agglutinin, for example, may be taken up by nerve terminals and retrogradely transported to the CNS by axons.	[184]
	Receptor-mediated transcytosis	Where nanoparticles with various ligands on their surfaces can bind to certain receptors and so enhance endocytosis GLUT1, lactoferrin (Lf), transferrin (Tf), or other peptides such as angiopep-2 or Seq12 have all been employed as targets.	[185,186]
Endocytosis	Clathrin-mediated endocytosis	Endocytotic vesicles up to 200 nm in diameter are formed in clathrin-enriched portions of the cell membrane. Once within the cell, clathrin-coated vesicles fuse together to form an early endosome, which progresses to late endosomes when the intravesicular pH drops and, eventually, lysosomes, triggering cargo destruction. To be an effective delivery agent, the nanoparticle must make it easier for its payload to escape from endosomes before they merge with lysosomes, preventing cargo destruction.	[187]
	Caveolin-mediated endocytosis	This occurs in lipid rafts and results in plasma membrane invaginations of roughly 80 nm in size. Following this first stage, caveolin vesicles merge with other caveolin vesicles to form caveosomes, which evade lysosomes and have a variety of fates depending on the cell type.	[187]

**Table 3:** Examples of clinical trials investigating BBB-related conditions.

Study Title	Intervention/Treatment	Status (as Reported until 15 April 2022)	ClinicalTrials.gov Identifier
Blood–Brain Barrier Permeability Study in Adults with Meningitis (NM-BBBP)	Other: ICG-PULSION Device: LiMON, Pulsion Medical Systems Other: Gadolinium (Gadovist, Bayer, Germany)	Terminated	NCT02902588
The Relevance of the Blood–Brain Barrier to Cognitive Dysfunction and Alzheimer’s Disease	Diagnostic Test: Contrast agent enhanced MRI using Gadovist	Completed	NCT04093882
Blood–Brain-Barrier Opening Using Focused Ultrasound with IV Contrast Agents in Patients With Early Alzheimer’s Disease (BBB-Alzheimers)	Device: BBB opening	Completed	NCT02986932
Surgical Tissue Flap to Bypass the Blood Brain Barrier in GBM	Procedure: Tissue autograft of pedicled temporoparietal fascial (TPF) or pericranial flap to bypass the blood brain barrier (BBB)	Recruiting	NCT03630289
Laparoscopically Harvested Omental Free Tissue Autograft to Bypass the Blood Brain Barrier (BBB) in Human Recurrent Glioblastoma Multiforme (rGBM)	Procedure: Laparoscopically harvested omental free flap	Recruiting	NCT04222309
Blood–Brain Barrier Opening Using MR-Guided Focused Ultrasound in Patients with Amyotrophic Lateral Sclerosis	Device: Blood–Brain Barrier opening with MRgFUS	Active, not recruiting	NCT03321487
The Level of Blood–Brain Barrier Damage Biomarker in Acute Ischemic Stroke	Diagnostic Test: the level of biomarker in blood	Recruiting	NCT05321225
Non-invasive Blood–Brain Barrier Opening in Alzheimer’s Disease Patients Using Focused Ultrasound	Device: Neuronavigation-guided single-element focused ultrasound transducer Drug: Definity Other: Magnetic Resonance Imaging (MRI) with or without gadolinium contrast agents Other: Positron Emission Tomography (PET) Other: Amyvid	Recruiting	NCT04118764
Blood–Brain Barrier Penetration of Therapeutic Agents in Human (BRIAN)	Drug: ODM-104 Drug: Paracetamol	Completed	NCT04571996
A Study to Evaluate Temporary Blood–Brain Barrier Disruption in Patients with Parkinson’s Disease Dementia	Device: MR Guided Focused Ultrasound Blood Brain Barrier Disruption using FUS Other Name: ExAblate	Active, not recruiting	NCT03608553
ExAblate Blood–Brain Barrier Opening for Treatment of Alzheimer’s Disease	Device: Blood Brain Barrier (BBB) Disruption	Recruiting	NCT03739905
ExAblate Blood–Brain Barrier (BBB) Disruption for the Treatment of Alzheimer’s Disease	Device: Blood Brain Barrier (BBB) Disruption	Recruiting	NCT03671889
Blood Brain Barrier Dysfunction and Postoperative Neurocognitive Disorders (BBBSx)	Diagnostic Test: Brain Imaging Diagnostic Test: Cognitive Testing Diagnostic Test: Blood Biomarkers	Recruiting	NCT04566562
Ultrasound-based Blood–Brain Barrier Opening and Albumin-bound Paclitaxel for Recurrent Glioblastoma (SC9/ABX)	Device: Sonication for opening of blood–brain barrier Drug: Chemotherapy, albumin-bound paclitaxel	Recruiting	NCT04528680
Safety Study of the Repeated Opening of the Blood–Brain Barrier with the SonoCloud^®^ Device to Treat Malignant Brain Tumors in Pediatric Patients (SONOKID)	Device: SonoCloud^®^ (9 transducers)	Not yet recruiting	NCT05293197
Developing Advanced Blood–Brain Barrier Permeability Imaging for Early AD	Device: GRASP DCE-MRI Device: 3T Brain Scan	Recruiting	NCT03389698

## Data Availability

Not applicable.
